# fMRI Activity in Posterior Parietal Cortex Relates to the Perceptual Use of Binocular Disparity for Both Signal-In-Noise and Feature Difference Tasks

**DOI:** 10.1371/journal.pone.0140696

**Published:** 2015-11-03

**Authors:** Matthew L. Patten, Andrew E. Welchman

**Affiliations:** 1 School of Psychology, University of Birmingham, Edgbaston, United Kingdom, B15 2TT; 2 School of Psychology, UNSW Australia, Sydney, NSW, Australia; 3 Department of Psychology, University of Cambridge, Cambridge, United Kingdom, CB2 3EB; University Medical Center Groningen UMCG, NETHERLANDS

## Abstract

Visually guided action and interaction depends on the brain’s ability to (a) extract and (b) discriminate meaningful targets from complex retinal inputs. Binocular disparity is known to facilitate this process, and it is an open question how activity in different parts of the visual cortex relates to these fundamental visual abilities. Here we examined fMRI responses related to performance on two different tasks (signal-in-noise “coarse” and feature difference “fine” tasks) that have been widely used in previous work, and are believed to differentially target the visual processes of signal extraction and feature discrimination. We used multi-voxel pattern analysis to decode depth positions (near *vs*. far) from the fMRI activity evoked while participants were engaged in these tasks. To look for similarities between perceptual judgments and brain activity, we constructed ‘fMR-metric’ functions that described decoding performance as a function of signal magnitude. Thereafter we compared fMR-metric and psychometric functions, and report an association between judged depth and fMRI responses in the posterior parietal cortex during performance on both tasks. This highlights common stages of processing during perceptual performance on these tasks.

## Introduction

The successful use of visual information relies on the brain’s ability to extract meaningful targets from cluttered backgrounds. This skill depends on (i) detecting and segmenting visual elements into coherent targets, and (ii) discriminating the diagnostic features of different target objects. For instance, a hiker on an arid trail would want to (i) break the camouflage of a snake from surrounding rocks and (ii) determine whether it is a rattlesnake or harmless gopher snake. These processes are central to everyday visual function, yet the neural architecture that supports them is not fully understood.

**Fig 1:**
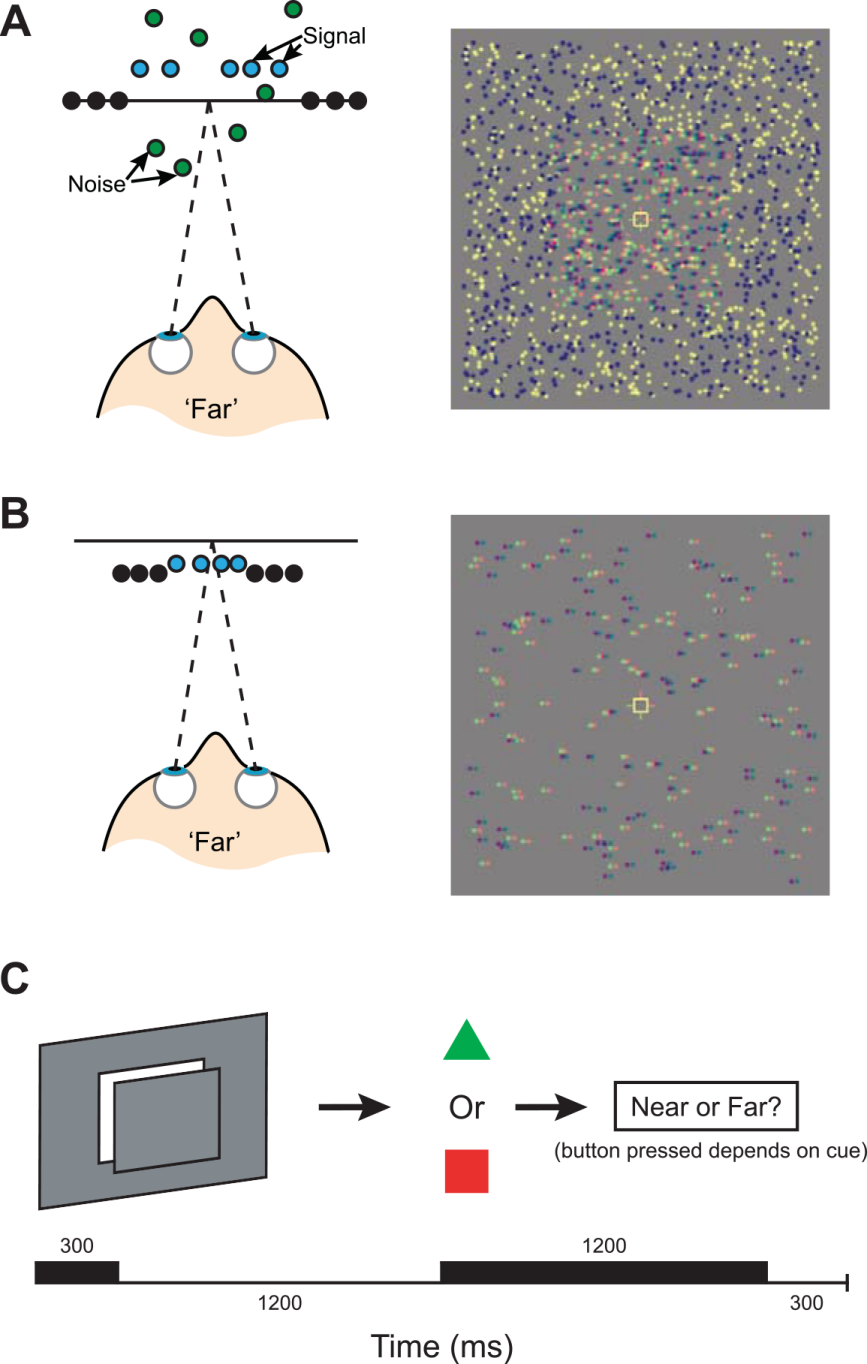
Stimuli and design of signal-in-noise and feature difference tasks. **(A)** A representation of stimuli in the signal-in-noise task viewed from above, and an illustrative red-green stereogram of the stimulus. Only a certain percentage of dots are located at the correct depth plane; the remainder are given random disparities. In the left image, blue dots represent ‘signal’ dots that were located at the target disparity, green dots represent distracting ‘noise’ dots, and black dots represent the surrounding pedestal disparity, located at the plane of fixation (horizontal line). In the experiment, all dots were black or white. **(B)** A representation of stimuli in the feature difference task viewed from above, and an illustrative stereogram of the stimulus. The pedestal was always located at a crossed disparity and the central test plane was titrated around this. Although all dots are given a crossed disparity, there is a central patch of dots that have a smaller disparity and appear behind the surrounding dots; therefore this stimulus is classified as ‘far’. **(C)** The procedure for a typical trial. The first black bar indicates stimulus onset and duration, the second black bar indicates presentation of the response cue.

It is believed that binocular disparity between the two eyes plays a significant role in both breaking camouflage to segment objects, and providing fine structural shape information that differentiates objects. Studies of disparity processing in the primate brain have examined the processes of segmentation and discrimination by contrasting performance on two different types of task. In particular, signal-in-noise (or “coarse”) tasks ask an observer to detect a disparity-defined target plane hidden in a cloud of random dots that mask the target, while feature difference (or “fine”) tasks require an observer to judge small differences between the depth of a target and its local neighborhood (**Fig 1**).

There is evidence that these tasks rely on different neural substrates: area MT/V5 appears important for signal-in-noise tasks, while V4 has been implicated in feature difference tasks. In particular, for a signal-in-noise task, there were similarities between neural and psychophysical sensitivity in macaque MT/V5 [1,2], with electrical stimulation there biasing psychophysical judgments [3] and inactivation reducing perceptual performance [4]. By contrast, electrical stimulation of MT/V5 had no measurable effect on a feature difference task [5]. Considering activity in V4, performance on a feature difference task was affected by microstimulation [6] and neurons in both V4 and later ventral regions (inferior temporal cortex, IT) showed activity during the fine task that predicted the perceptual choice of the monkey [6,7].

This work points to a task-based dissociation between MT/V5 and V4; nevertheless, it is quite likely that there are common stages of processing for both tasks. In particular, theoretical models propose that both types of task engage (i) the readout of feature representations and (ii) external noise exclusion [8–10].

Here we sought to use fMRI to measure the circuits involved during performance of both signal-in-noise and feature difference tasks. Based on the evidence reviewed from electrophysiological recordings in MT/V5 *vs*. V4, we might expect to observe different circuits engaged by the two tasks: dorsal areas may be particularly engaged by the signal-in-noise task, while ventral activity may be critical for the feature difference task. To look for similarities between perceptual judgments and fMRI responses, we adapted approaches that link neuronal responses and behavioral performance (e.g., ‘neurometric functions’, [1,2,5,11,12]). In particular, we used information decoding to assess whether aggregated patterns of brain activity contain information about presented stimuli in a manner that is similar to behavioral performance ('fMR-metric functions' [13]). To this end, we rendered depth planes in random-dot stereograms (RDS) and sought to manipulate participants’ behavioral ability to judge depth position (near *vs*. far) in one of two ways (**Fig 1A and 1B**). In Experiment 1, we varied the percentage of dots defining the position of the target plane in relation to dots with randomly assigned disparities (i.e., we varied the signal-to-noise ratio). In Experiment 2, we titrated the disparity difference between a target plane and its surround. We considered fMRI responses in independently-defined regions of interest in the visual and parietal cortices, and use multi-voxel pattern analysis (MVPA) to determine the extent to which near *vs*. far depth positions could be decoded based on the fMRI activity. We assessed changes in classification performance and behavioral performance as the signal strength was manipulated, to test for areas whose activity changes in a manner that is similar to changes in perceptual judgments [11,14].

## Materials and Methods

### Participants

21 participants from the University of Birmingham were recruited for both signal-in-noise (n = 10; 3 females) and feature difference (n = 11; 5 females) experiments. Mean age was 26.2 years (range, 19–35 years). All participants had normal or corrected-to-normal vision with no deficits in color vision and were paid for their participation. All participants were screened for stereo deficits in the laboratory to ensure they could discriminate at least 1 arcmin of disparity. Seven further participants were tested, but subsequently excluded from further analysis. Five were excluded due to excessive head movement during scanning (their head moved more than 4 mm or 4 degrees drift over the course of the scan) which disrupts the voxel co-registration logically required for the MVPA decoding technique. The other two participants were excluded due to poor behavioral performance in the scanner (did not sample a psychometric function across tested conditions with near-chance performance across all conditions except for the 100% signal condition). The study was approved by the University of Birmingham STEM ethical review committee and all participants provided written informed consent.

### Stimuli

We developed stimuli for the signal-in-noise task and feature difference task that sought to ensure that similar disparity magnitudes were presented for both (see also [15,16]). Previous electrophysiological work using these tasks generally employed different disparity magnitudes (± 2 deg *vs*. ± 6 arcmin, hence the terms “coarse” and “fine”), as well as different types stimulus manipulations (signal-to-noise ratio *vs*. disparity titration). It is known that disparity magnitude can affect fMRI responses [17–21], so we therefore sought to test of a similar range for both tasks, well within the “fine” disparity range [22].

#### Experiment 1: Signal-in-noise task

We used random-dot stereograms (RDS) defined by black and white dots within a rectangular aperture (14 x 19°) and displayed on a mid-grey background. Within this region, a central test plane (7 x 7°) was given a nonzero disparity of ±6 arcmin relative to the fixation plane. To minimize the effects of adaptation across trials, some jitter (up to ±1 arcmin) was added to the disparity value for each trial. The dot density of the stereogram was 8 dots/deg^2^ and each dot in the stereogram had a Gaussian luminance profile with a diameter (at half-height) of 0.15°. The stereogram region was surrounded by a grid of black and white squares which were used to provide an unambiguous background reference and promote stable vergence throughout the experiment. A fixation marker was presented in the center of the stimulus that consisted of a hollow white square (0.5° side length), with horizontal and vertical nonius lines (length 0.375°) to assist in maintaining eye vergence at the fixation target (in the plane of the screen). We restricted presentation of dots to outside of a circular region (1.5° diameter) centered at fixation to reduce interference from the stimulus on binocular fusion. Monocular zones were controlled by shifting dots that overlapped with the surround RDS to the opposite side of the target plane so the RDS had a consistent size for all disparities. We manipulated task difficulty by introducing noise dots that were located at random depths, up to a maximum of ±20 arcmin (**Fig 1A**). In the 0% signal condition, there was no consistent depth plane and all dots appeared scattered in depth, while in the 100% signal condition no noise dots were presented. We employed five different signal levels (0, 20, 40, 60 and 100%) to sample different levels of psychophysical performance. Under all conditions, dots outside the target were located at the plane of the screen and contained no disparity noise.

#### Experiment 2: Feature difference task

We presented RDS consisting of two concentric squares (side lengths 7° and 14°) at different depths. Task difficulty was manipulated by titrating the disparity of the target with respect to the surrounding pedestal (**Fig 1B**). The pedestal had a crossed disparity of 12 arcmin and the target plane varied ±6, 18, 30, 60 or 240 arcsec around this. Some jitter (up to ±1 arcmin) was added to the disparity value of both planes for each trial. Our pilot psychophysical testing revealed that observers were too sensitive to the disparity differences we might reasonably display for the dense random dot patterns, we therefore reduced the dot density to 1 dot/deg^2^ to ensure the task was sufficiently difficult. We excluded dots in the neighborhood of the fixation marker (circular exclusion zone 2° in diameter) to minimize interference with binocular fusion. Since both planes had nonzero disparities, we removed the entire RDS for the fixation condition, displaying only the fixation marker and background.

### Design

Psychophysical and fMRI data were collected concurrently from each observer in a single session of approximately nine event-related scans. On each trial, participants made a perceptual judgment on the depth sign of the stimulus (i.e., ‘near’ or ‘far’). The order of trials was matched for history (one trial back), such that each trial was equally likely to be preceded by any of the conditions. The order of the trials differed across runs and participants. Eleven conditions were presented on each run: 10 stimulus conditions (5 signal levels × crossed *vs*. uncrossed disparities) and one fixation condition during which the target plane was removed. We collected 11 repetitions of each trial type on each run (total 121 trials) and added a single dummy first trial to ensure that trial history of the second trial was balanced. Each scan started and ended with a 9 s fixation interval, and total duration of a single scan was 6 min 24 s.

Experimental trials lasted 3 s (**Fig 1C**) and started with 300 ms stimulus presentation followed by a delay of 1200 ms (75% of trials) or 1400 ms (25% of trials) during which the central test plane was removed from the screen. These different delay times were chosen to minimize predictability and habitual responding by participants. To measure perceptual judgments, while dissociating the motor response (button press) from the perceptual interpretation, we used a delay-cue response paradigm. In particular, after the variable delay, a green triangle or red square appeared inside the fixation marker and served as an indicator for the motor response mapping to be used on that trial. If the response cue was a green triangle, observers used a particular finger-key matching (e.g., index finger for ‘near’), while if the response cue was a red square, observers switched finger-key matching (e.g., index finger for ‘far’). This was balanced across participants to remove any bias for a particular cue. The response cue was removed 300 ms before the next trial onset. During fixation trials, the fixation square and surround RDS were simply displayed for 3 s.

### Laboratory testing prior to fMRI

Participants were familiarized with both the tasks and the delay-cue response task in the laboratory prior to scanning for an average of 2.4 hrs. All participants bar one had past experience of psychophysical testing.

### fMRI data acquisition

The study was performed in a 3-Tesla Philips Achieva MRI scanner at the Birmingham University Imaging Centre. Echo-planar imaging (EPI) and T1-weighted anatomical (1 x 1 x 1 mm) data was collected using an eight-channel SENSE head coil. For both experiments, EPI data [echo time (TE), 35 ms; repetition time (TR), 1500 ms] were acquired from 25 slices (voxel size, 2 mm isotropic, near coronal; 256 volumes) covering the visual cortex, posterior parietal cortex and posterior temporal cortex. Localizers were obtained in a separate session, with EPI data (TE, 34 ms; TR, 2000 ms) acquired from 28 slices (voxel size, 1.5 x 1.5 x 2 mm, near coronal). Stereoscopic stimulus presentation was achieved using a pair of video projectors (JVC D-ILA SX21), each containing separate spectral comb filters (INFITEC, GmBH) whose projected images were optically combined using a beam-splitter cube before being passed through a wave guide into the scanner room. The INFITEC interference filters produce negligible overlap between the emission spectra for each projector, meaning that there is little crosstalk between the signals presented on the two projectors for an observer wearing a pair of corresponding filters. Stimuli were projected onto a translucent plastic screen behind the head coil inside the bore of the magnet. Participants viewed the screen (optical path length, 65 cm) through a mirror positioned on the head coil, angled at 45°. Unique stimuli were pre-generated for each participant using C^#^, and the experiment was controlled using MATLAB (The MathWorks, Natick, MA) and the PsychToolBox 3 extension [23,24].

**Fig 2:**
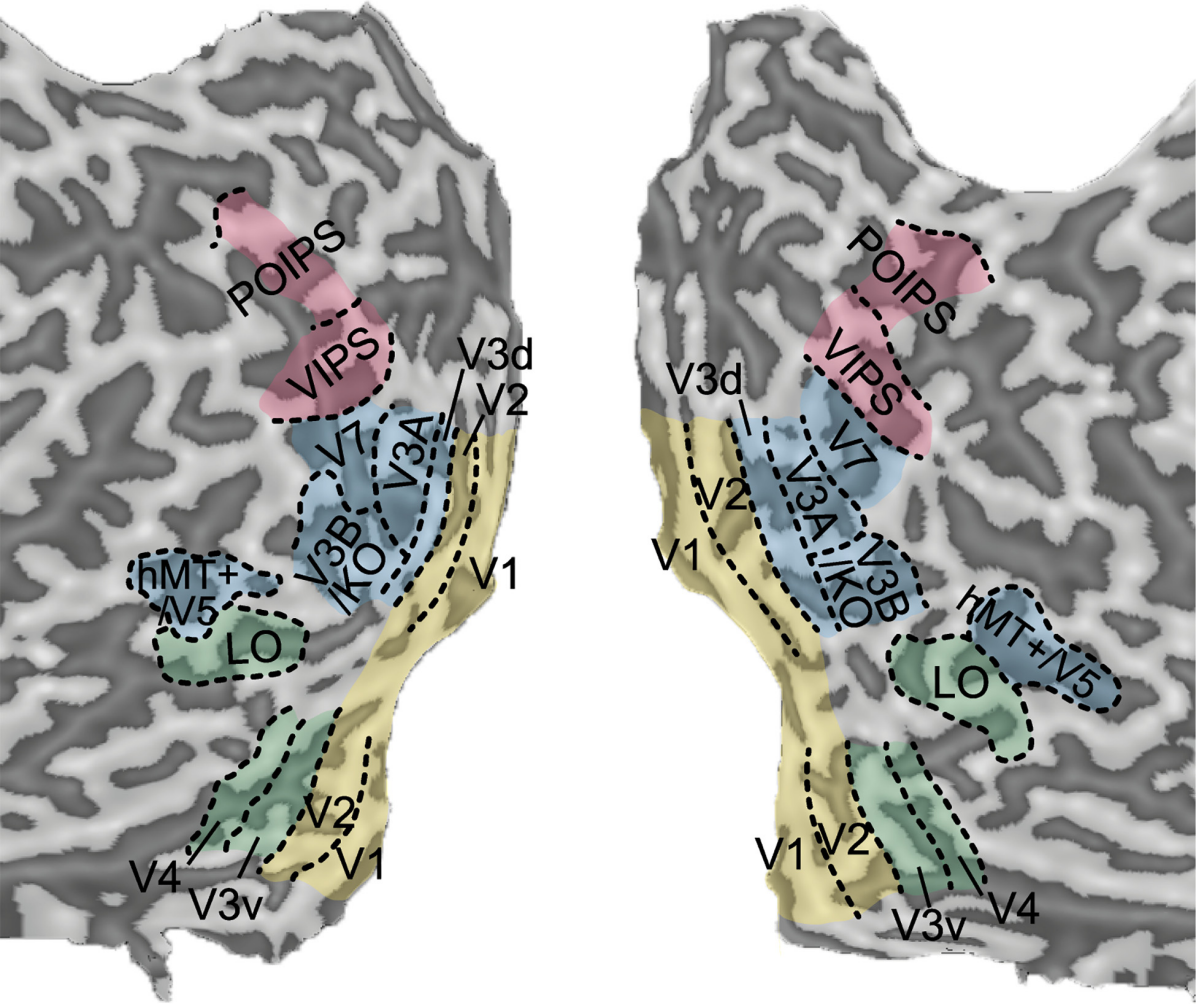
Regions of interest for a single subject. Regions of interest in a representative participant showing the retinotopic areas, V3B/KO, hMT+/V5, LO and parietal regions VIPS and POIPS. Regions were defined using independent localizers. Sulci are coded in darker gray than the gyri.

For each participant, we identified regions of interest (ROIs) using standard mapping techniques. This approach allowed us to localize ROIs based on independent data from the data collected during the experimental trials. The borders of early retinotopic regions (V1, V2, V3v, V4, V3d, V3A and V7) were localized using rotating wedge stimuli and expanding concentric rings [25–27]. In particular, V4 was defined as the region of retinotopic activation in ventral visual cortex adjacent to V3v that contained a representation of the upper visual field [28–29]. V7 was defined as a region anterior and dorsal to V3A [29–31]. In addition, we identified higher dorsal regions: V3B/kinetic occipital area (KO), human motion complex (hMT+/V5) and the ventral lateral occipital region (LO) using independent localizer scans. Area V3B/KO [32–33] was defined as the region of cortex with a full hemifield representation located inferior to, and sharing a foveal representation with, V3A [29]. This retinotopically defined area overlapped with the set of contiguous voxels that responded significantly more (*p* < 10 ^− 4^) to kinetic boundaries than transparent motion of a field of black and white dots. Area hMT+/V5 was defined as the region in the lateral temporal cortex that responded significantly higher (*p* < 10 ^− 4^) to an array of inward and outward coherently moving dots than to an array of static dots [34]. The lateral occipital (LO) region was identified as anatomically posterior portion of the region active in the lateral occipito-temporal cortex that was significantly more responsive (*p* < 10 ^− 4^) to intact rather than scrambled images of objects and shapes [35,36]. During the localizer scans, participants performed an attentionally demanding task on the fixation point, except for the lateral occipital (LO) localizer in which they had to respond if the same image was presented consecutively. For all participants in the feature difference task and two participants in the signal-in-noise task, regions along the intraparietal sulcus [ventral IPS (VIPS); parieto-occipital IPS (POIPS)] were identified by contrasting the activity from three-dimensional shape (defined by both disparity and structure-from-motion cues) to random patterns (shuffled disparities and motion speeds) [14,37]. For remaining participants, these regions were defined as the area anterior to V7 which showed significantly stronger (*p* < 10 ^− 4^) responses to all of the experimental conditions in contrast to the fixation baseline. We were unable to obtain data for the parietal region POIPS for two participants due to the spatial resolution of the EPI sequence and near coronal slice positioning during the fMRI acquisition. For illustrative purposes, the mapping of these ROIs for one participant is presented in **Fig 2**.

### fMRI data analysis

MRI data were processed using BrainVoyager QX (BrainInnovation, Maastricht, the Netherlands). For each subject, anatomical scans were transformed into Talairach space and used for 3D cortex reconstruction, inflation, flattening and the segmentation of gray and white matter. Preprocessing of functional data included three-dimensional correction of head movements, slice scan time correction, temporal high-pass filtering (3 cycles per run cutoff) and removal of linear trends. Functional runs were aligned using the anatomical data as a positional reference and transformed into Talairach space.

We used Multi-Voxel Pattern Analysis (MVPA) software to analyze the fMRI data from each ROI. Specifically, we used a linear support vector machine derived from SVM^light^ binaries [38] with a linear kernel function and a cost parameter set as the inverse of the sum of all training data squared and divided by the number of cross-validations. For voxel selection, gray matter voxels were isolated through a gray-matter mask from the anatomical scan and sorted according to their response (*t* statistic) to all stimulus conditions compared with a fixation baseline across all experimental runs. Using the *t* values from this “all stimuli versus fixation baseline” contrast, we restricted the pattern size to those voxels that showed a value larger than 0, and selected the 300 most active voxels (or highest number available) in each ROI for use in the classification. Examining prediction accuracies across pattern size (number of voxels) showed that classification values had saturated by 300 voxels in all of the ROIs we considered. (See Preston *et al*. [21] for a detailed treatment that quantifies pattern-based decoding for different numbers of voxels within an ROI and comparing between different ROIs). Estimation of fMRI responses to single events in our event-related fMRI design were likely to be noisy for single trials; therefore, prior to feeding the data to the machine learning classifier we averaged a small number of trials (4, 4 and 3 from a single run) to generate 3 training patterns per run [13]. Each voxel’s time series was then normalized (*z*-score) in each experimental run. The fMRI time series was shifted by 3 volumes (4.5 s) to account for the hemodynamic delay of the BOLD signal. Each volume had the mean univariate signal subtracted from it.

To evaluate the performance of the MVPA classification algorithm, we used a leave-one-run-out cross-validation analysis procedure. For each cross-validation, one run was left out as an independent test dataset and the data from the remaining runs was used as the training set. This resulted in 56 training patterns and 6 test patterns per signal level. The prediction accuracy for each ROI was obtained by averaging the prediction accuracy across cross-validations. Training was performed on the condition with highest signal (i.e., 100% correlated for signal-in-noise task; 240 arcsec disparity difference for the feature difference task) since conditions with higher external or internal noise are likely to increase variability and provide training data that is less reliable. We then calculated test patterns for all five signal levels. The calculated prediction accuracy of the classifier corresponds to the proportion of trials on which the algorithm could correctly predict the stimulus based on the pattern of fMRI responses, in which chance performance would be 0.5 for a binary classification (i.e., ‘near’ *vs*. ‘far’ stimuli). It is important to note that the voxel selection (i.e., based on independent localizer scans and stronger responses to all stimuli *vs*. fixation) was independent of the classification contrasts (i.e., near *vs*. far). For each region of interest, prediction accuracies were averaged across participants.

A standard cumulative Gaussian function was used to fit both the behavioral results and prediction accuracies for each ROI using a procedure that implemented a maximum likelihood method [39]. The cumulative Gaussian was generated by computing threshold and slope parameters while restricting the bounds of the function through guess and lapse rate parameters. To estimate the extent to which decoding accuracies might arise by chance, we trained the classifier on data from the highest signal condition with shuffled stimulus labels and repeated this for 999 bootstrapped resamples. The lower bound of the fMR-metric functions (0% signal condition for the signal-in-noise task, or a depth separation of 0 arcsec for the feature difference task) was restricted to lie within the 32^nd^ and 68^th^ centiles (i.e., +/- one standard error) of the shuffled distribution, so that all fits were restricted to originate within the bounds of chance performance. Further, we scaled the cumulative Gaussian model by restricting the mean and threshold parameters to those from the psychophysical data and fit the fMRI data using a least squares estimation. Finally, we tested the fit of the scaled fMR-metric function to the fMRI data points in each ROI by measuring the goodness-of-fit at corresponding values using the coefficient of determination.

## Results

### Signal-in-noise task

**Fig 3:**
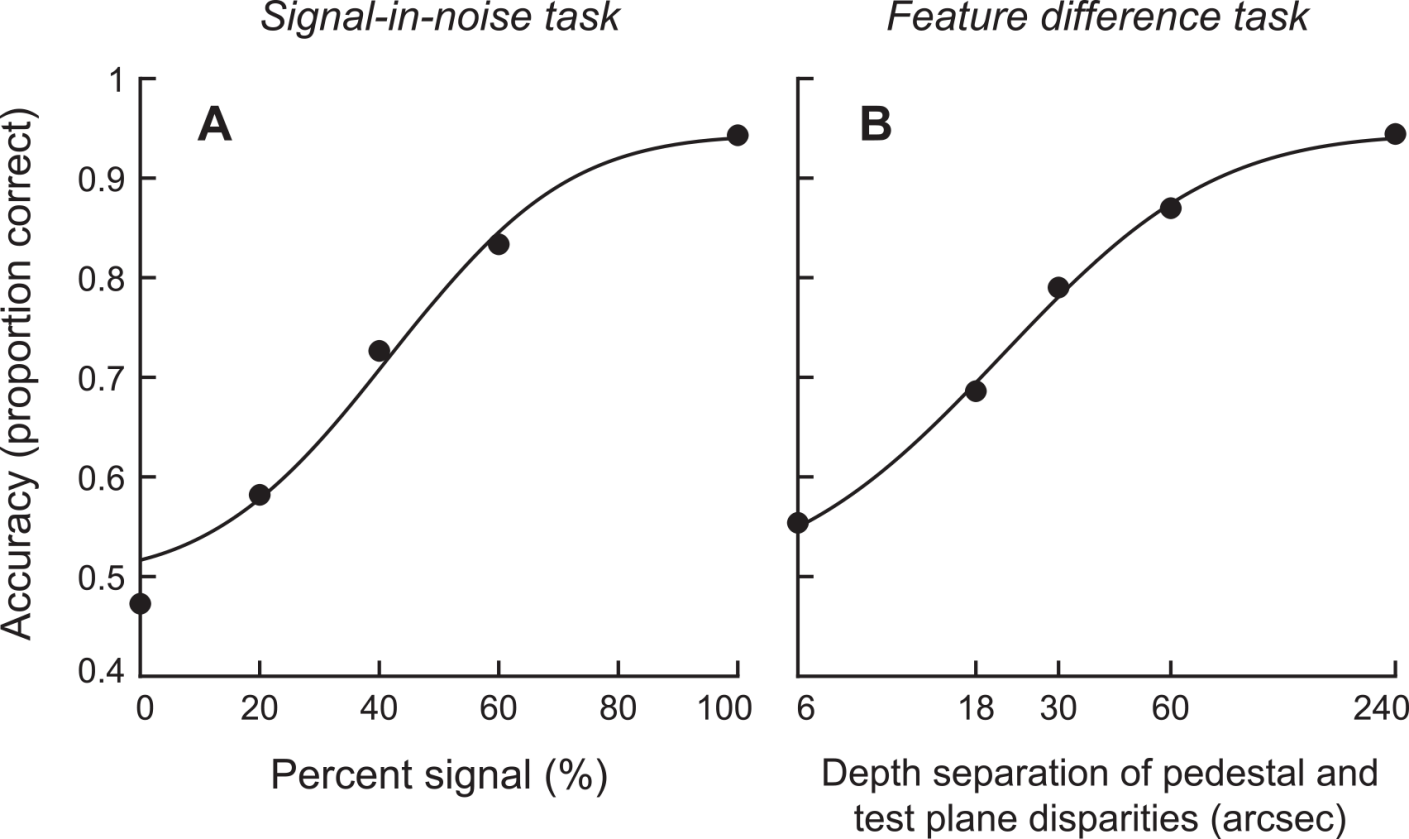
The mean behavioral results for both experiments. **(A)** Behavioral results for the signal-in-noise task, where the signal reflects the percentage of dots that were at the disparity of the test plane and accuracy refers to the proportion of responses that the participant correctly identified as near or far. The solid curve is the best-fitting Gaussian. **(B)** A similar function for the behavioral results for the feature difference task placed on a logarithmic scale.

During the signal-in-noise task, participants discriminated the depth position (closer or farther than the fixation plane) of a central target while we measured fMRI responses in regions of interest in the visual and parietal cortices. We manipulated the difficulty of the behavioral task by changing the proportion of signal dots in the central target relative to noise dots that had a randomly chosen disparity. As the proportion of noise dots increased―and fewer signal dots were present―the task became increasingly difficult. Based on pilot testing, we selected five different signal levels to use during fMRI scanning that sampled different locations on the psychometric function. To describe psychophysical performance, we fit the behavioral judgments with a cumulative Gaussian (**Fig 3A**). We measured the 75% threshold of this function to find that on average, participants needed 42% signal dots to judge the depth position of the target. This value is somewhat higher than those measured in macaque monkeys (typically between 10 and 20% [1,2]) however our stimuli had a smaller range of disparities and our presentation was much briefer (300 *vs*. 1500 ms).

We examined fMRI responses sampled from early visual areas (V1, V2), ventral regions (V3v, V4, LO), dorsal regions (V3d, V3A, V3B/KO, V7, hMT+/V5) and parietal regions (VIPS, POIPS). Using the measured fMRI responses, we trained a linear support vector machine to associate patterns of voxel activity within each region of interest (ROI) to the disparity-defined depth position of the stimulus that gave rise to the activity. We tested whether we could predict the viewed stimulus from the fMRI activity, calculating the mean leave-one-run-out prediction accuracy for classifiers trained to discriminate crossed from uncrossed disparities when no noise was present.

**Fig 4:**
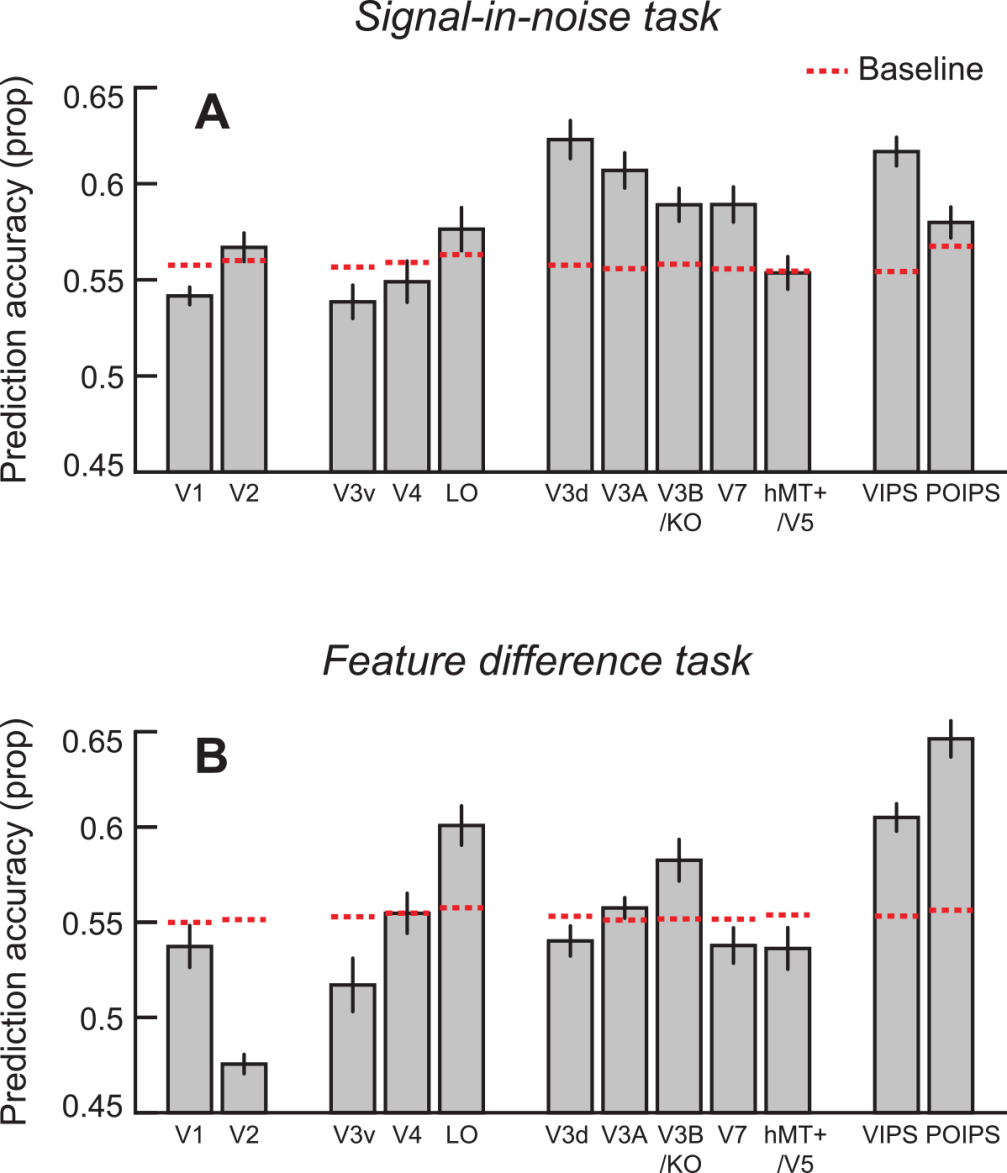
Prediction accuracies for each region of interest. **(A)** The mean prediction accuracy of the classifier for the 100% signal condition in the signal-in-noise task. The horizontal red lines mark the baseline of statistical significance generated from permuting the data labels before being fed into the classifier. The location of the line indicates the upper 99.5% centile of the distribution of permuted data. **(B)** The mean prediction accuracy of the classifier for the 240 arcsec condition in the feature difference task. Again, the dotted horizontal lines indicate the cut off for statistical significance based on a permutation analysis. Error bars depict the SEM.

**Fig 4A** shows the between-subjects mean prediction accuracies obtained for the most discriminable stimulus configurations (100% signal) for each ROI. To establish a baseline for chance performance, and thereby judge responses that were statistically reliable, we ran the classification analysis with randomly permuted fMRI patterns (i.e., we randomized the correspondence between fMRI data and training labels and estimated the classifier prediction for each visual area) over 999 bootstrap iterations for the 100% signal condition. This created a distribution of classification accuracies, and we used the upper 99.5^th^ centile (one-tailed, Bonferroni corrected) as our criterion for statistical significance (**Fig 4**, dotted lines). For all regions of interest, the median of the shuffled distribution was very close to 0.5 (range, 0.498–0.501) confirming our analysis technique to be unbiased. Considering responses across the sampled regions of interest, we were not able to reliably decode near *vs*. far depth differences in early visual area V1, ventral regions V3v and V4 or dorsal region hMT+/V5 based on activity measured using the event related fMRI design. However, we found that measured fMRI responses supported classification accuracies that exceeded the criterion for chance decoding in early visual area V2, ventral region LO and for all of the remaining measured dorsal and parietal visual areas.

**Fig 5:**
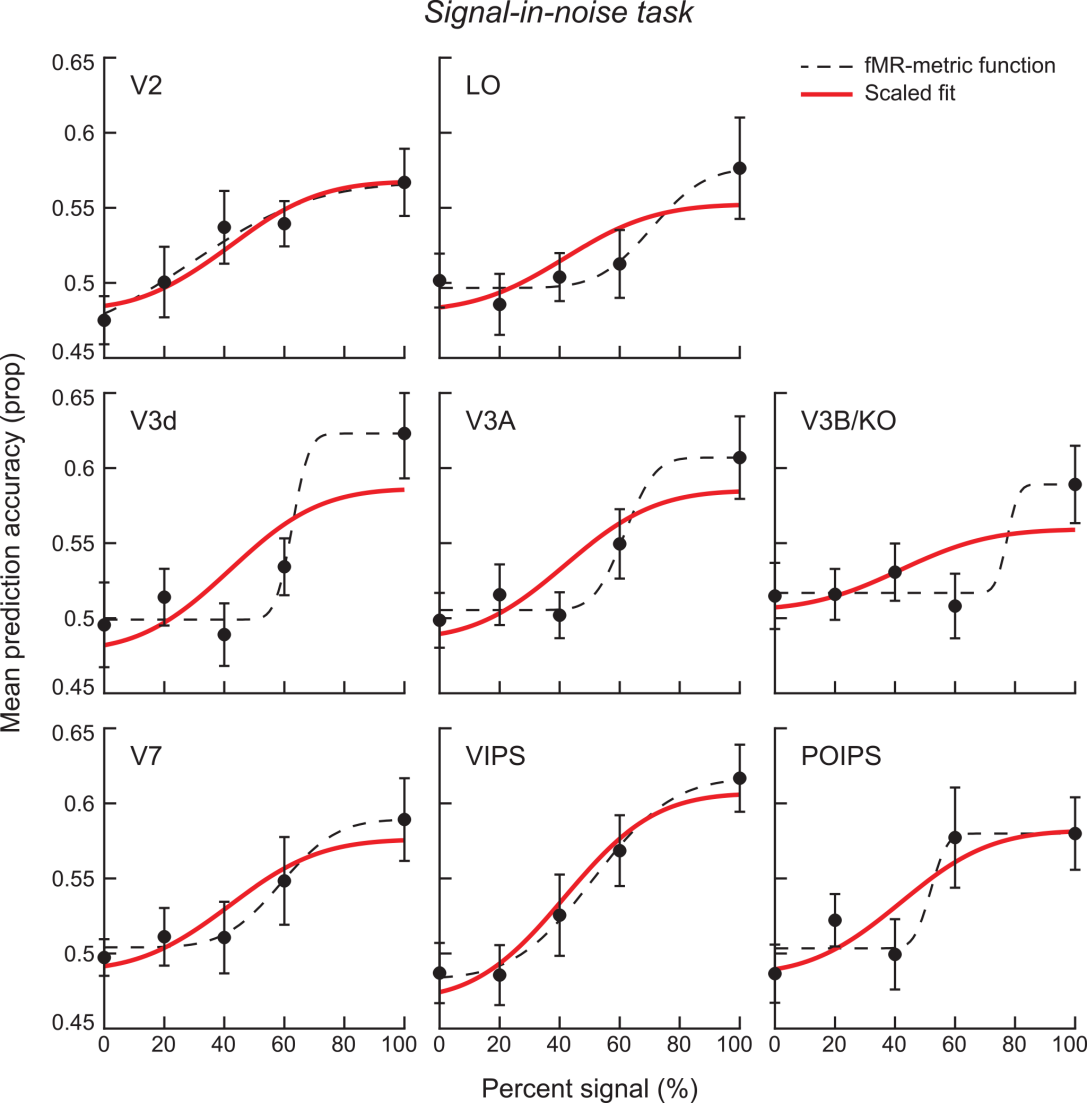
fMR-metric functions for the signal-in-noise disparity task. The red line is a scaled version of the behavioral results and the dashed line is the best-fitting Gaussian. Error bars on each datum show the between-subjects SEM.

To test for similarities between behavioral judgments of depth position and information about depth position contained in the fMRI responses, we evaluated the decoding performance of the classifier at different signal levels in the regions of interest in which we could reliable decode the stimulus for 100% signal. Thereby, we generated an 'fMR-metric' function [13], which we then fit using a cumulative Gaussian (**Fig 5**, dashed lines). To compare our ability to decode depth positions from participants’ fMRI activity with the behavioral performance of the participants, we used the parameters (mean, threshold) of the psychometric function (**Fig 3A**) to constrain the Gaussian fit to the fMRI data, allowing the maximum value to vary as a free parameter while the minimum was constrained to be near chance (i.e., within one standard error of mean classifier performance when data labels had been permuted). This created a scaled version of the behavioral results and allowed us to compare the simultaneously recorded fMRI activity and behavioral performance. We present these fMR-metric functions for ROIs with above chance performance in **Fig 5**and performed a goodness-of-fit test to quantify the fit of the fMRI classification accuracies to the values on the scaled fMR-metric function (**Table 1**). fMRI responses in V2 as well as intermediate dorsal (V3A, V7) and early parietal (VIPS, POIPS) visual areas could be decoded in a manner that was similar to the behavioral performance. In contrast, we did not observe a parametric effect in earlier dorsal regions V3d (*R* = .80, *p* = .104), V3B/KO (*R* = .65, *p* = .232) or in ventral region LO (*R* = .81, *p* = .094), with performance in these regions found to deteriorate as soon as noise was introduced into the stimulus.

**Table 1:** Goodness-of-fit of the fMR-metric functions for the signal-in-noise task. A goodness-of-fit test of the fMRI data points for the signal-in-noise disparity task (**Fig 5**) to the scaled version of the behavioral results (**Fig 3A**).

	Pearson Corr	P-value
**V2**	.958	**.010**
**LO**	.813	.094
**V3d**	.800	.104
**V3A**	.879	**.049**
**V3B/KO**	.654	.232
**V7**	.936	**.019**
**VIPS**	.981	**.003**
**POIPS**	.885	**.046**

These results suggest similarity between perceptual judgments of depth position in a signal-in-noise task and fMRI responses in a number of visual areas: V2, V3A, V7, and most strongly in VIPS and POIPS. In contrast, introducing noise dots to the display appears to severely disrupt our ability to decode near *vs*. far depth positions in dorsal regions V3d, V3B/KO and also in higher ventral region LO. The finding of activity in dorsal areas is compatible with previous results from dorsal area MT/V5 in suggesting a link to performance on a signal-in-noise task [1–3,5]. Decoding performance in area hMT+/V5 was unfortunately not reliably above chance performance, making it difficult to draw a direction comparison between species. This is likely due to the absence of motion from our stimuli, and compatible with our recent work using rTMS during viewing of these stimuli [16].

### Feature difference task

The results of Experiment 1 indicated that we could decode depth positions from ventral area LO under 100% signal conditions, but performance deteriorated rapidly once a small amount of noise was introduced. In Experiment 2, we tested performance in the feature difference task that does not involve adding noise perturbations to the display but is instead limited by the participants’ ability to differentiate stimulus features (i.e., depth position) based on the precision of their internal representation. In particular, participants judged small differences in the relative disparity between a target plane and its surround (which was located in front of the fixation plane). For this stimulus, all of the dots in the stimulus carried signal. Our principle interest was in whether a different set of areas would be found to be important for the performance of this task, with an *a priori* expectation that ventral circuits might be more critical.

Participants were asked to decide whether the central presented plane was in front or behind the surrounding pedestal reference plane. We considered five different disparity separations, selected so as to characterize variations in psychophysical performance similar to those measured in Experiment 1. We fit the behavioral results using a cumulative Gaussian (**Fig 3B**) and measured the 75% threshold of this function to find that participants required around 21 arcsec disparity difference to judge the target’s position relative to the surround. This is within the range of values observed for macaque monkeys performing a similar task [5,6].

**Fig 6:**
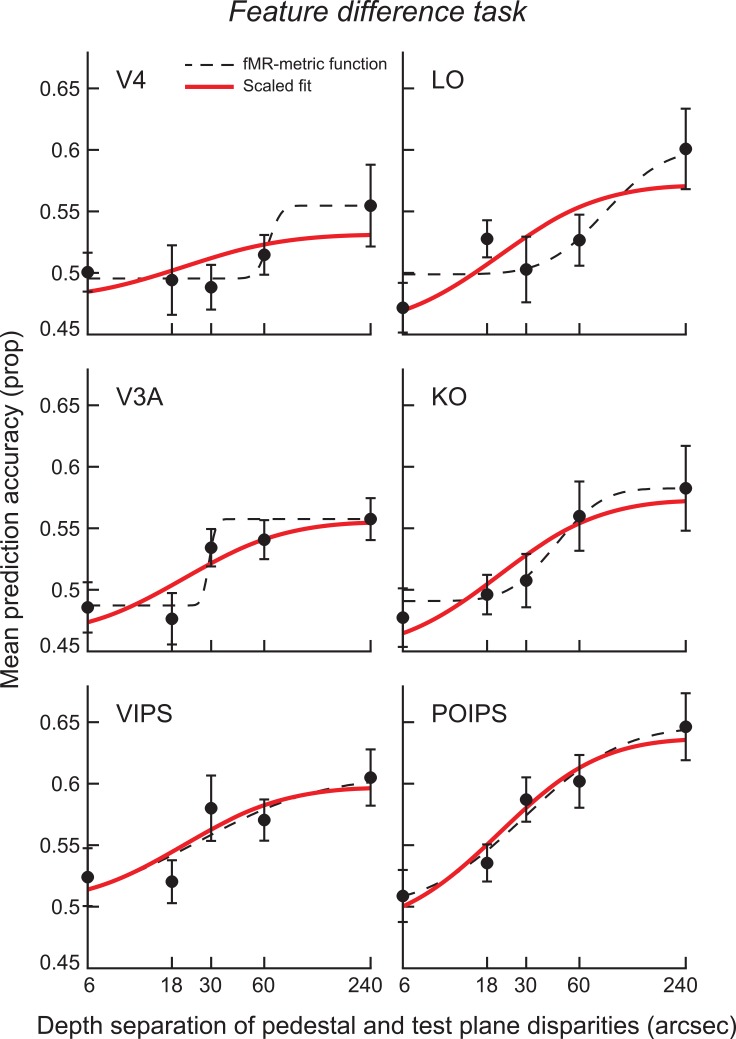
fMR-metric functions for the feature difference task. The red line is a scaled version of the behavioral results and the dashed line is the best-fitting Gaussian. Error bars on each datum show the between-subjects SEM.

Following the analysis approach used in Experiment 1, we first tested for areas in which we could reliably decode relative depth positions for the highest signal levels. We found above chance decoding performance in ventral areas V4 and LO, and dorsal areas V3A, V3B/KO, VIPS and POIPS (**Fig 4B**). Thereafter, we considered the changes in prediction accuracies for regions of interest in which we could reliably decode stimuli in the clearest difference conditions. In particular, we examined the performance of the classifier as the disparity difference between the target and its surround was varied, and computed fMR-metric functions by fitting a cumulative Gaussian sigmoid to the fMRI decoding data (**Fig 6**, dashed lines). In addition, we used the psychometric function to fit the fMRI data, restricting the shape of the function to that of the behavioral results and constraining the minimum to be within one standard error of the shuffled distribution (for a disparity difference of 0 arcsec) while allowing the maximum to vary as a free parameter.

Testing the goodness-of-fit showed significant fits of the behavioral results to the fMRI data in dorsal (V3A, V3B/KO) and parietal (VIPS, POIPS) regions, while a marginal fit was observed for ventral area LO (*R* = .83, *p* = .080) (**Table 2**). In particular, activity in parietal regions VIPS and POIPS showed the clearest similarities to depth judgments for the feature difference task and largely replicated our finding from the signal-in-noise task. Responses in other dorsal areas (V3A, V3B/KO) and ventral area LO were also reasonably well described by the behavioral model; however these regions supported lower prediction accuracies that were close to chance levels, meaning that our detection power was limited.

**Table 2:** Goodness-of-fit of the fMR-metric functions for the feature difference task. A goodness-of-fit test of the fMRI data points for the feature difference task (**Fig 6**) to the scaled version of the behavioral results (**Fig 3B**).

	Pearson Corr	P-value
**V4**	.682	.204
**LO**	.833	.080
**V3A**	.891	**.043**
**V3B/KO**	.948	**.014**
**VIPS**	.881	**.048**
**POIPS**	.976	**.005**

### Control analyses

**Fig 7:**
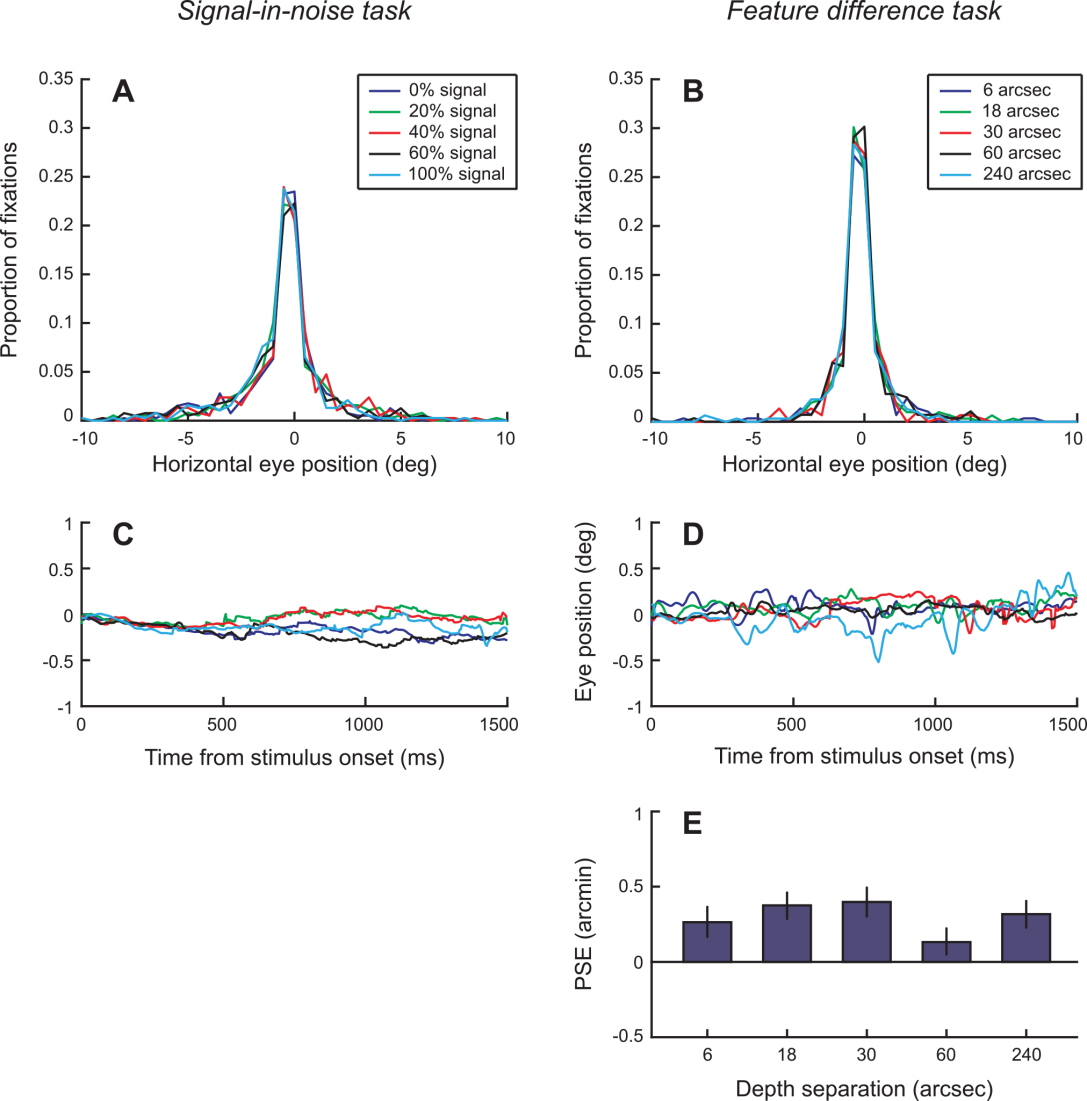
Analysis of eye-movements and vernier task performance. **(A, B)** The distribution of horizontal eye positions in relation to the fixation marker are shown for (A) the signal-in-noise task and (B) the feature difference task. **(C, D)** Event-related eye position traces in the different conditions for the signal-in-noise task (C) and feature difference task (D). **(E)** The points of subjective equality for behavioral performance on the vernier task. Error bars are the standard deviation confidence limits of the psychometric fit.

We took a number of precautions to avoid experimental artifacts and ensure that our data treatment was appropriate. First, we recorded horizontal eye-movements for participants in both experiments using a monocular limbus eye-tracker (CRS ltd). Analyses of these data suggested no systematic difference in eye-position (**Fig 7A–7D**) and no statistical difference in the number of saccades between conditions. The confines of the scanner and the use of spectral filters for stereoscopic presentation meant that we were not able to measure eye vergence objectively (the monocular limbus tracker is the only system compatible with the binocular setup we use). However, we designed our stimuli with the aim of reducing the likelihood of vergence changes: participants were instructed to use the horizontal and vertical nonius lines to assist them in ensuring correct eye alignment at all times, and we surrounded the stimuli using a stable, low spatial frequency pattern in the plane of the screen. Additionally, for the signal-in-noise task the central test plane was surrounded by a zero-disparity RDS. However, for the feature difference task the pedestal RDS was presented at a crossed disparity on all trials and it is possible that participants’ vergence state changed and became biased away from the fixation plane. To address this, before scanning commenced all participants were presented with each stimulus condition randomly for a total of 200 trials while participants undertook a vernier task that provided a subjective measure of vergence state [40]. We found that there was little bias in the psychometric functions obtained from the vernier task, and no systematic differences between conditions (**Fig 7E**). This suggests that observers were able to maintain stable vergence across conditions (perhaps assisted by the background reference marks and the fixation point) despite the vergence demand of the pedestal plane near the point of fixation.

A potential concern in relation to fitting fMR-metric functions to the data from the feature difference task is that, unlike the signal-in-noise task, the upper disparity value we chose (4 arcmin) does not provide a natural ceiling for performance on the task. In particular, while perceptual judgments have saturated by this point, we could not be certain that fMRI responses might not become more discriminable had larger disparities been presented. We therefore sought to test whether decoding accuracies would continue to increase with the presentation of larger disparities. In particular, three participants returned to take part in an additional session of the feature difference experiment using larger depth separations between the pedestal and test planes (2, 4, 6, 8, 10 arcmin). We found that MVPA accuracies did not change systematically as disparity was increased, and formally tested the relationship between disparity magnitude and prediction accuracy using regression analyses. The slope parameter was not reliably different from zero (based on 95% confidence intervals), suggesting that under the event-related fMRI paradigm accuracies had saturated at the point where the stimuli were perceptually highly discriminable. In particular, for both VIPS (-.024, .005) and POIPS (-.021, .019) the estimated slope of the regression line was not different from zero. Thus, the decoding accuracies obtained during the main experiment were saturated when participants were engaged in online feature difference judgments. This confirmed that we had appropriately sampled the upper bound of the fMR-metric function and that, like psychophysical performance, increasing disparities further resulted in performance at ceiling.

Finally, it is unlikely that changes in prediction accuracies obtained using measurements from parietal cortex could be related to task difficulty rather than stimulus processing *per se*. In particular, the MVPA technique we used relies on discriminative differences between conditions (i.e., crossed *vs*. uncrossed disparity at the same signal level), so attentional differences would not be expected between different disparity signs at the same signal level. Second, our use of a delayed cue paradigm that decoupled the motor response from the perceptual interpretation required that participants maintain task engagement during the delay period, minimizing the potential for differences in attentional allocation for the different signal levels.

## Discussion

We investigated fMRI responses to disparity-defined stimuli while participants performed different perceptual tasks (signal-in-noise and feature difference tasks) that have previously been found to have different neuronal bases. We used a parametric fMRI approach that was coupled with online psychophysical judgments to test for similarities between perception and brain activity at different stimulus presentation levels. We found we were able to decode clearly-defined depth differences in both ventral and dorsal cortical areas; however, the relationship between the decoding of these signals and changes in the perceptual output was strongest in higher dorsal stream areas. In particular, our results suggest a close association between fMRI activity and human depth discrimination in the later regions of the dorsal pathway (V7, VIPS and POIPS) for the signal-in-noise task, and in early parietal regions VIPS and POIPS for the feature difference task.

### Task-related activity in later dorsal regions

The relationship we observed between changes in fMRI activity and perceptual judgments (i.e., fMR-metric *vs*. psychometric functions) in regions of the intraparietal sulcus (VIPS, POIPS) suggest that these areas are at a high level within the chain of computations and complete depth judgments based on information collected across regions of the visual cortex. Previous work has indicated that regions V7, VIPS and POIPS contain a registered depth impression as they are activated by a variety of depth configurations, including planar surfaces [21,31], 3D curvature [14,20,41], depth positions and depth structure [42]. This is compatible with a generalized representation of disparity-defined depth; a suggestion that is further supported by responses in the parietal regions of the macaque. Single unit recordings from the caudal intraparietal area (CIP) have shown responses to surface structures defined by both binocular disparity [43–47] and monocular depth cues (e.g., perspective, texture, motion) [46–49]. Comparisons between monkey and human fMRI suggest that CIP could be the homologue of VIPS [31,42], or both VIPS and POIPS [48]. Moreover, single unit responses in lateral (LIP) and anterior (AIP) intraparietal areas have revealed responses to depth structure and 3D shape [50–52]. Further, responses to disparity-defined objects in the AIP were absent for anticorrelated stimuli [52] suggesting the representation of disparity-defined stimuli that rejects false-matches based on a contrast-similarity constraint, consistent with the perceptual interpretation of such stimuli (*cf*. [21]). These generalized responses are therefore compatible with our evidence that fMRI activity in later dorsal regions (particularly VIPS and POIPS) reflects a higher stage of disparity processing that is closely associated with an individual’s 3D perceptual interpretation.

It is most likely that the responses we observe in VIPS and POIPS reflect a read-out signal in both tasks, where these computed depth signals are used to decide on the depth position which is then forwarded for functional use. In the context of a motion signal-in-noise tasks, LIP neurons have been shown to be predictive of an animal’s perceptual decision [53]. Further, evidence from an ambiguous apparent motion task suggests that neuronal responses in monkey LIP vary in line with the animal’s perception [54].

### Interpreting results from other regions of interest

Responses in the primary visual cortex did not exceed the shuffling distribution for either task (**Fig 4**) and this is consistent with the notion that this area relates to the processing of local disparity signals rather than the global percept [14,21,55–57]. In subsequent region V2 we observed a significant fit of the fMR-metric function to the psychophysical results for the signal-in-noise task, though prediction accuracies for this region were close to the threshold for statistical significance (see **Fig 4A**). This may be due to V2 being involved in more elementary processes such as figure-ground segregation and the processing of disparity edges [58–61], since the disparity difference between target and surround planes were the inverse of one another for ‘near’ and ‘far’ stimulus conditions.

While we were able to decode depth positions in dorsal areas (V3d, V3A, V3B/KO) as well as ventral area LO, we did not obtain strong evidence for an association between psychometric and fMR-metric decoding based performance in these areas. Nevertheless, such a null result does not rule out the involvement of these areas in task performance. Namely, a prediction accuracy result below the shuffled distribution does not in itself imply that a region is not involved as it is possible that our data acquisition and/or analysis methods were insufficiently sensitive to detect contributions from these regions. In particular, an essential component of our paradigm was to measure behavioral performance concurrent to fMRI responses. This required an event-related design in which conditions varied trial-by-trial. Such designs are known to produce noisier estimates due to the successive presentation of different conditions before the hemodynamic response has returned to baseline [62]. While we took measures to reduce the impact of this noise (counterbalancing conditions, pooling activation from trials prior to classification), this design reduced the sensitivity with which we could decode depth for both tasks. Specifically, the highest prediction accuracies we obtained were in the region of 65% which is considerably below the accuracies of up to 85% observed elsewhere using similar stimuli in a blocked fMRI design [21,63].

Another point of consideration is the ease of interpreting a significant goodness-of-fit statistic of the psychophysical function to the fMRI data when prediction accuracies are near chance. Although prediction accuracies in the lower signal conditions are expected to be located at, or close to chance by nature of the paradigm, it is possible that random fluctuations can result in a significant fit of the fMR-metric function. Obtaining more sample points on the fMR-metric functions could have helped to address this issue, but this was not possible within the fMRI design constraints of collecting sufficient stimulus repetitions for the MVPA. We minimized the potential for random responses using a criterion that performance in the highest signal case should exceed 99.5% of the accuracies obtained with randomly permuted data in our analysis. However, some regions had prediction accuracies close to chance for all lower signal levels and still contained significant or marginal fits to the scaled behavioral results (for instance, area V3A for the feature difference task; **Fig 5**). Finally, while previous neurophysiology studies have compared the responses of single neurons with the behavioral performance of the monkey [1–5,7], here we computed fits using the between-subjects mean. This approach is common when using fMRI measures because of the inherent variability of the measurements, particularly in relation to the event-related design.

### The effect of training

Recent human psychophysical work with disparity-defined signal-in-noise and feature difference tasks has demonstrated interactions in the transfer of training effects between signal-in-noise and feature difference tasks [15], and there is growing evidence that identified substrates for task performance can be altered by training. In particular, Chowdhury and DeAngelis [4] established that a period of training on a feature difference task changed the functional importance of MT/V5 for a signal-in-noise task: before training MT/V5 inactivation disrupted performance, but not after training. Following up on this work, Chang et al [16] used human transcranial magnetic stimulation to show a functional reorganization of processing in higher-level visual areas as a result of training. Specifically, they reported that the effects of dorsal stream stimulation were diminished by training (stimulation in posterior parietal cortex affected signal-in-noise task performance before, but not after, feature difference training), while ventral area LO became important for the signal-in-noise task as a result of training (LO stimulation affected performance after, but not before, training).

In our study, participants underwent minimal training before being scanned and only for the purpose of familiarization of the tasks and delay-cue response procedure. The exposure provided to test stimuli prior to scanning was unlikely to be enough training to elicit reorganization of cortical areas relating to the two tasks and our results are likely to reflect that of pre-training performance. Nonetheless, we cannot exclude the possibility that familiarization on these tasks beforehand may have affected some level of change in the neural response.

### The relationship to relative disparity processing

An alternative means of formulating the functional roles of the visual pathways relates to the type of disparity processing used, where specialization for relative disparity occurs in the ventral stream to aid computations of surface structure and 3D shape. Indeed, selectivity to relative disparity has been observed in ventral region V4 [6,64] and has not been found in the dorsal pathway when using a center-surround configuration [5,65]. Our feature difference task required comparing the relative depth of the target and surround planes, and thus this viewpoint suggests computation would take place in ventral regions. Similarly, the signal-in-noise task could, in principle, be solved using absolute disparities (though relative disparities were still present in that the fixation marker and surround plane were displayed throughout all trials) and would be predicted to take place in the dorsal stream. However, our results suggest the involvement of dorsal and parietal regions in the completion of both tasks.

### Disparity magnitude

For both experiments, we presented stimuli that contained disparities in the classically-defined ‘fine’ range. In particular, our feature difference task used a minimum relative disparity of 6 arcsec to a maximum of 4 arcmin (an absolute disparity of 16 arcmin when including the pedestal depth), while our signal-in-noise task was located at ±6 arcmin disparity. The transition between ‘fine’ and ‘coarse’ disparities has been suggested to occur at a magnitude greater than 20–30 arcmin [22,66]. While our design is consistent with the disparity magnitudes in previous work that employed feature difference (“fine”) tasks [4–6], there was a clear difference in that our signal-in-noise (“coarse”) task used disparities with smaller magnitudes than previously employed (typically 30 arcmin or more): [1–5]. It is possible that our limited detection power in, for instance, hMT+/V5 relates to the smaller disparity magnitudes we have employed. This might interact with the absence of motion from our stimuli: for instance, MT/V5 neurons show weaker responses to binocular disparity when using static stimuli [67].

It is also important for us to note that while the magnitudes of disparities were similar for our two tasks, it was necessary for us to modify dot density to bring performance on the feature-difference task to comparable levels with the signal-in-noise task. This difference in stimulus parameters represents a complicating factor in contrasting task performance that was an unavoidable necessity.

### Understanding disparity organization

Understanding the processing hierarchies for disparity processing in the primate brain is a considerable challenge, and there are good reasons to posit different computations and selectivities between streams in terms of their ultimate functional goals of action control and recognition. One framework for thinking about disparity processing is that there is a distinction in the magnitude of disparity that each visual pathway is thought to compute [68]. As reviewed in the Introduction, there is considerable electrophysiological evidence to posit differential involvement of MT/V5 *vs*. V4 during the performance of signal-in-noise and feature difference disparity tasks. Yet our fMRI results suggest the common involvement of posterior parietal circuits during performance on both types of task. This is likely to relate to the accumulation of sensory evidence towards a perceptual decision. Recent TMS work suggested dissociable contributions of posterior parietal cortex and ventral area LO in performance of these tasks. However, the current results indicate this dissociation is likely to reflect different stages of estimation and can be optimized through training [16] rather than a strict dissociation between functional loci.

In summary, we simultaneously measured fMRI activity and behavioral performance on two different depth discrimination tasks and used a multivariate classifier to identify cortical regions that related to task-dependent use of disparity. We found higher dorsal regions showed responses that were decoded in line with the perceptual use of disparity signals for both tasks. In particular, a close association was found for V7, VIPS and POIPS in the signal-in-noise task (containing noise dots), and for VIPS and POIPS during the feature difference task (without noise dots). These higher stages of processing are likely to be involved reading out feature representations and accumulating evidence while making perceptual decisions.
